# Human Umbilical Cord Mesenchymal Stem Cells Prevent Steroid-Induced Avascular Necrosis of the Femoral Head by Modulating Cellular Autophagy

**DOI:** 10.3390/biomedicines12122817

**Published:** 2024-12-12

**Authors:** Changheng Zhong, Hanzhe Xu, Junwen Chen, Wenxiang Cai, Jianlin Zhou, Hao Peng

**Affiliations:** Department of Orthopedics Surgery, Renmin Hospital of Wuhan University, Wuhan 430060, China; 18706827939@163.com (C.Z.); 2018283020157@whu.edu.cn (J.C.); 2024283020153@whu.edu.cn (H.X.); 2020283020214@whu.edu.cn (W.C.)

**Keywords:** glucocorticoid-induced osteonecrosis of femoral head, human umbilical cord mesenchymal stem cell, autophagy, core decompression, osteoblasts

## Abstract

Background: Glucocorticoids (GCs) are critical regulatory molecules in the body, commonly utilized in clinical practice for their potent anti-inflammatory and immunosuppressive properties. However, prolonged, high-dose GC therapy is frequently associated with femoral head necrosis, a condition known as glucocorticoid-induced osteonecrosis of the femoral head (GC-ONFH). Emerging evidence suggests that enhanced autophagy may mitigate apoptosis, thereby protecting osteoblasts from GC-induced damage and delaying the progression of ONFH. This study aims to evaluate whether human umbilical cord mesenchymal stem cells (hUCMSCs) can alleviate GC-induced osteoblast injury through autophagy modulation. Methods: In vitro, osteoblasts were exposed to GCs for 48 h, followed by co-culture with hUCMSCs for an additional 12 h before further analysis. The osteoblasts were categorized into four experimental groups: (A) control group, (B) Dex group, (C) Dex + hUCMSC group, and (D) Dex + hUCMSC + 3-MA group. In vivo, rabbits were assigned to one of four groups: Con, MPS, core decompression (CD), and CD + hUCMSC (*n* = 12 per group), and subsequently subjected to CT imaging and HE staining. Results: In vitro results demonstrate that hUCMSC treatment mitigated GC-induced osteoblast apoptosis and preserved osteogenic activity through autophagy modulation. In vivo, infusion of hUCMSCs enhanced trabecular thickness in the femoral head and improved the femoral head microenvironment. Conclusions: These findings suggest that hUCMSCs protect osteoblasts from GC-induced damage by regulating autophagy, offering new insights into the potential therapeutic use of hUCMSCs for treating ONFH via autophagy enhancement.

## 1. Introduction

Steroid-induced avascular necrosis of the femoral head (SANFH) results from prolonged or high-dose glucocorticoid (GC) use, leading to impaired blood supply to the femoral head, ischemia-driven degeneration and death of osteoblasts, and ultimately, collapse of the femoral head [1,2]. GCs are recognized as the primary cause of non-traumatic osteonecrosis of the femoral head [3]. The disease typically progresses insidiously, with early symptoms being subtle and often unnoticed. As the condition advances, joint pain and restricted mobility become prominent, significantly impairing patients’ functional capacity and quality of life. Consequently, SANFH is considered one of the orthopedic conditions with the highest disability rates [4,5]. Current early-stage interventions, including the use of fibrinolytics, lipid-lowering agents, anticoagulants, and vasodilators, are insufficient to halt lesion progression, despite demonstrating some clinical benefit [4,6]. As the disease progresses to advanced stages, surgical intervention becomes necessary, though it is costly, invasive, and prone to complications. Thus, there remains an urgent need for safer and more effective early treatment options [7,8].

Recent studies have highlighted the significant potential of mesenchymal stem cell (MSC)-based therapies in slowing the progression of various diseases [9,10,11]. MSCs, which possess multidirectional differentiation capabilities [12,13,14], are a class of adult stem cells that include those derived from synovial tissue, adipose tissue, and bone marrow [15]. Among these, human umbilical cord mesenchymal stem cells (hUCMSCs) stand out due to their distinct advantages over other stem cell types, such as lower transplantation-related mortality (TRM) and a reduced risk of disease recurrence following transplantation [16]. These properties have garnered increasing attention and have driven extensive research. Furthermore, the umbilical cord, routinely discarded as medical waste after childbirth, represents an easily accessible and ethically uncontroversial source of stem cells, avoiding the moral concerns associated with alternative stem cell sources.

Further investigation is required to fully elucidate the pathological role of GCs in SANFH. As research advances, increasing focus has been placed on the critical roles of cellular autophagy and apoptosis in the disease’s progression. Autophagy, a lysosome-mediated self-degradation process, occurs in eukaryotic cells and can be classified into three types: macroautophagy, microautophagy, and chaperone-mediated autophagy (CMA), with macroautophagy being the most widely studied [17]. This tightly regulated process plays a key role in maintaining cellular homeostasis by facilitating the degradation of damaged organelles, misfolded or unfolded proteins, and pathogens. Autophagic vesicles encapsulate these materials, which are then transported to lysosomes for degradation, providing essential energy for the cell [18]. Similarly, apoptosis, another vital cellular process, refers to programmed cell death controlled by genetic factors. It serves as a spontaneous mechanism that helps organisms respond to external stresses, ultimately facilitating adaptation to their environment [19]. The interplay between autophagy and apoptosis is complex, with significant overlap in their regulation and outcomes [20]. Recent studies have shown a correlation between autophagy and the pathogenesis of SANFH [21]. Autophagy influences SANFH in multiple ways, though the exact nature of its interaction with the disease remains a subject of debate. Autophagy is widely regarded as having a dual role, exhibiting both protective and detrimental effects depending on the cellular context and environmental factors [22]. Therefore, precise modulation of autophagy may offer a promising therapeutic strategy for mitigating the progression of SANFH, potentially shifting the paradigm of its treatment.

The development of SANFH is influenced by the balance between osteoblast and osteoclast activity [23]. Dexamethasone (DEX), a widely used glucocorticoid, primarily targets osteoblasts, where it can trigger apoptosis through the activation of the autophagy pathway [24]. DEX induces autophagy, which helps to preserve osteogenic function following GC exposure by interacting with the apoptosis-regulatory proteins Bax and Bcl2 [25]. This suggests that autophagy serves as a pro-survival response to DEX, mitigating its toxic effects and maintaining cell viability. In the autophagic signaling pathway, FIP200 deficiency impairs the formation of the FIP200-ULK1-ATG13 (FIP200-Unc-51 Like Autophagy Activating Kinase 1-autophagy related 13 Gene) complex, inhibiting autophagy in both osteoblasts and osteoclasts, leading to increased apoptosis and disrupting the osteoblastic/osteoclastic balance. This highlights the protective role of autophagy in the pathogenesis of ONFH [26]. Experimental data from Wang XY et al. demonstrated that pine membrane proteins activate autophagy and reduce GC-induced apoptosis and bone thinning by inhibiting the PI3K/Akt/mTOR pathway [27]. Estrogen has also been shown to enhance autophagy and inhibit osteoblast apoptosis through the ER-ERK-mTOR pathway [28]. Abnormalities in cellular autophagy are linked to a range of diseases, including amyotrophic lateral sclerosis, Alzheimer’s disease, and other neurodegenerative conditions [29]. Recent studies have suggested that MSCs can modulate autophagy to exert therapeutic effects [30]. For instance, MSCs have been shown to repair atherosclerosis-induced myocardial injury by enhancing autophagy levels [31] and to reduce amyloid β-protein accumulation, improving memory deficits [32]. However, no study has yet demonstrated the potential of MSCs in modulating autophagy to protect against SANFH.

Autophagy and apoptosis share several common upstream signaling pathways, which link the two processes closely [33]. Typically, mild autophagy inhibits apoptosis, while increased apoptosis suppresses autophagy; excessive autophagy can exacerbate cellular damage by degrading intracellular organelles [34]. Autophagy inhibits apoptosis primarily through mitochondrial autophagy [35]. When mitochondria are damaged, proteins such as Bax and BH3-only proteins translocate, leading to mitochondrial outer membrane permeabilization (MOMP) [36]. MOMP results in the dissipation of mitochondrial transmembrane potential (ΔΨm) and the release of pro-apoptotic factors, triggering cell death. As a protective mechanism, autophagy is activated to degrade damaged mitochondria, thereby preventing apoptosis [37]. Beyond mitochondrial autophagy, autophagy also inhibits apoptosis by degrading key apoptosis-related proteins in the cytoplasm [38]. For instance, autophagy selectively removes activated caspase 8, thereby inhibiting TRAIL-induced apoptosis. Inhibition of autophagy, such as through Atg7 knockdown, leads to increased caspase 8 activity [39]. Thus, enhancing autophagy to clear damaged mitochondria appears to be an effective strategy to protect osteoblasts from GC-induced injury.

These findings provide a strong theoretical foundation for using hUCMSCs as adjunctive therapy in the early non-surgical treatment of GC-induced ONFH. By establishing a rabbit model of GC-induced ONFH and an in vitro DEX-induced osteoblast injury model, the potential role of hUCMSCs was evaluated. The results of our research demonstrate their protective effects on the femoral head microstructure, supporting the therapeutic potential of hUCMSCs in managing GC-ONFH.

## 2. Materials and Methods

### 2.1. Preparation and Characterization of hUCMSC and Osteoblasts

hUCMSCs, prepared and provided by Shenzhen Wingor Biotechnology Co., Ltd. (Shenzhen, China), were collected after repeated passages to the 3rd–5th passage and frozen for experimental use. Characterization of hUCMSCs was conducted using flow cytometry and cell differentiation assays. The experimental procedure for detecting surface markers of CD73, CD105, CD90, CD11b, CD19, CD34, and in CD45 (all from BD Biosciences Pharmingen, San Jose, CA, USA) was as follows.

Mouse PE-IgG isotype immunoglobulin was used as a negative control. Primary cells were digested and PBS was added to adjust the cell concentration to 1 × 10^6^/mL. Five μL of fluorescently labelled monoclonal antibodies CD73-FITC (561254), CD105-PE (562759), CD11b-PE (743977), CD34-PE (340441), CD45-FITC (340943), CD90-PE (328107), CD19-PE (392507), and HLA-DR (307603) were added to 200 μL of cell suspension, mixed, and then incubated for 30 min at room temperature and protected from light, and the expression of surface antigens was detected by LSR II flow cytometry (BD Biosciences, Franklin Lakes, NJ, USA) using FlowJo software (Tree Star, Inc., Ashland, OR, USA) to analyze the data.

For the experiments of adipogenesis, osteogenesis, and chondrogenesis, hUCMSC (P3) were cultured with adipogenic, osteogenic, and chondrogenic differentiation kits (Procell, Wuhan, China), respectively. hUCMSCs were maintained in 6-well plates with a concentration of 5 × 10^4^ cells per well in duplicate. Media was changed every two days. The differentiation of hUCMSCs into adipocytes, osteocytes, and chondrocytes was conducted according to the manufacturer’s instructions. After induction of differentiation, the cells were fixed with 4% paraformaldehyde in PBS for 10 min and stained for 20 min at room temperature with the following staining: Adipogenesis was verified with Oil Red-O (Procell) staining. The osteogenesis matrix calcification, which is present following osteogenesis, was visualized with Alizarin Red (Procell). Excess stain was removed by several washes with PBS, and the stained cells were visualized with an upright microscope (BX53, Olympus, Japan). The chondrogenic differentiation was induced in three dimensions by the manufacturer’s instructions. After being embedded in paraffin, the chondrospheres were sectioned and stained with Alcian Blue (Procell, Wuhan, China) for 30 min, rinsed with distilled water for 2 min, and rinsed with distilled water once. The images of chondrogenic staining were observed and evaluated under an upright microscope (BX53).

Primary osteoblasts were isolated as previously described [40]. Briefly, 1–2-day-old newborn Sprague-Dawley (SD) rats were used. The cranial skull was removed under aseptic conditions and washed three times with PBS containing antibiotics. Blood vessels, mucous membranes, and connective tissues were carefully removed, and the bone was cut into 1–3 mm pieces. The bone pieces were digested with 0.25% trypsin (Procell, Wuhan, China) at 37 °C for 30 min, after which the digestive fluid was discarded. The bone was then digested with 0.1% collagenase II (Sigma-Aldrich, St. Louis, MO, USA) at 37 °C for 1.5 h with gentle agitation. The digestion solution was collected and centrifuged at 1000 rpm (radius 8 cm) for 10 min to collect the osteoblasts. Osteoblast characterization was performed using an alkaline phosphatase (ALP) activity assay and Alizarin Red S staining. All animal procedures received approval from the Ethical Review Committee for Laboratory Animal Welfare of Wuhan University People’s Hospital (WDRM20220106).

### 2.2. Cell Culture and Processing

Osteoblasts were cultured in DMEM (Procell, Wuhan, China) supplemented with 10% fetal bovine serum (FBS, Procell, China) and collected by centrifugation at 1000 rpm for 10 min. The cells were cultured in DMEM with 10% newborn calf serum, at 37 °C with 5% CO_2_, with medium changes every 48 h, followed by changes every 3 days. The cells were passaged, and the 4th-generation cells were used for experiments. hUCMSCs were cultured in Ham’s F-12 medium (Procell, China) containing 10% FBS (Procell, China) and 1% penicillin–streptomycin (Gibco, Grand Island, NY, USA). Passage 3 hUCMSCs were cultured in Transwell inserts (Corning, New York, NY, USA) at a density of 5 × 10^3^/cm^2^ for 10–12 h prior to use. DEX (Sigma, St. Louis, MO, USA) was dissolved in DMSO under light protection in an ultra-clean bench and prepared as a 0.1 M stock solution, which was stored at −20 °C under light protection. Osteoblasts were exposed to DEX for 48 h, after which they were co-cultured with hUCMSCs for an additional 12 h before further analysis. The experimental groups were as follows: (A) control group (no intervention), (B) Dex group (induced with DEX), (C) Dex + hUCMSC group (induced with DEX and co-cultured with hUCMSCs for 12 h), and (D) Dex + hUCMSC + 3-MA group (induced with DEX and 3-MA, and co-cultured with hUCMSCs for 12 h). 3-Methyladenine (3-MA) was used to inhibit class III phosphatidylinositol 3-kinase, thereby preventing autophagosome formation in the early stages of autophagy.

### 2.3. SANFH Animal Model and Groupings

The rabbit SANFH model was established following a protocol outlined in the literature [41]. New Zealand White rabbits (male, 3 ± 0.5 kg) were obtained from Wuhan Institute of Biological Products (Wuhan, China). The experimental animals were maintained in a specific pathogen-free environment with appropriate temperature (22–26 °C), humidity (50–70%), and light (12 h light/12 h dark cycle). After an acclimatization period of one week to minimize animal stress, modeling was initiated. Lipopolysaccharides (LPS, 10 μg/kg) (Beyotime, Haimen, China) were injected intravenously through the ear margin on day 1. Methylprednisolone (MPS, 20 mg/kg) (Beyotime, China) was administered intramuscularly once daily for three consecutive days (days 2–4). Basic animal conditions were monitored, and follow-up assessments were carried out after 4 weeks. The SANFH model rabbits were randomly divided into four groups: (1) Con group (no intervention), (2) MPS group (induced with MPS), (3) core decompression (CD) group (induced with MPS and undergoing CD following successful modeling), and (4) CD + hUCMSC group (induced with MPS, undergoing CD, and receiving hUCMSC infusion following successful modeling) (*n* = 12/group). All animal surgeries were conducted in the fluoroscopic operating room, People’s Hospital of Wuhan University, Wuhan, China. In the CD + hUCMSC group, hUCMSCs were injected into the bone wax using a syringe needle to puncture the bone wax with 0.2 mL of a hydrogel (Polyether F127 diacrylate, F127DA, EFL, Suzhou, China) containing hUCMSCs slowly injected into the bone cavity. The hUCMSC concentration in the hydrogel was 5 × 10^6^ cells/mL. In the CD group, 0.2 mL of hydrogel was injected using the same method, but without hUCMSCs. The MPS group underwent only the CD procedure at the femoral head without further interventions. All animal experiments were conducted in accordance with the Regulations on the Management of Laboratory Animals, strictly following the 3Rs principle. All animal procedures received approval from the Ethical Review Committee for Laboratory Animal Welfare of Wuhan University People’s Hospital (WDRM20220106) and adhered to the ethical guidelines set forth in the 8th edition of the Guide for the Care and Use of Laboratory Animals (National Research Council, Rockville, MD, USA, 2011).

### 2.4. Cell Viability Assay

Cell viability was assessed using the Cell Counting Kit-8 (CCK-8, Beyotime, Haimen, China) assay according to the instructions provided by the manufacturer. Cells were inoculated in 96-well plates, and different concentrations (50 μM, 100 μM, 150 μM, 200 μM, and 300 μM) of Dex were added. Three replicated wells were set up for each group. The cells were cultured at 37 °C for 12 h, 24 h, or 48 h. The supernatant was removed after centrifugation and 100 μL of fresh medium containing 10 μL of the CCK-8 solution were added to each well and incubated at 37 °C for 1 h. Cell viability was determined by measuring absorbance at 450 nm using a Tecan Sunrise microplate reader.

### 2.5. Flow Cytometry

An Annexin V-FITC/PI assay was used for determining osteoblast apoptosis. The cells were seeded into a 24-well plate with 3 × 10^4^ cells per well and then treated by Dex for 24 h. They were incubated at 37 °C. After modeling, the cells were centrifuged at 1000 r/min for 5 min, collected, washed with PBS, resuspended at 1–5 × 10^5^, added to 500 μL 1x binding buffer, and stained with 5 μL Annexin V-FITC (AV) and 10 μL propidium iodide (PI) for 20 min at room temperature, protected from light. Samples were then analyzed using a flow cytometer (BD, Influx), and CytExpert 2.0 software (Beckman Coulter, Lane Cove, NSW, Australia) was used for determining apoptotic cell percentage. Annexin V+/PI− cells were designated as early apoptotic cells, while Annexin V+/PI+ cells were identified as late apoptotic cells. The total percentage of apoptotic cells was calculated through the addition of the percentage of early and late apoptotic cells.

### 2.6. Cell Proliferation Assay

For osteoblast proliferation assays, osteoblasts were seeded in 6-well plates at a density of 2 × 10^5^ cells per well. Cell proliferation was assessed using BrdU (5-bromo-2-deoxyuridine) incorporation (Cell Signaling Technology, Danvers, MA, USA).

### 2.7. Alkaline Phosphatase (ALP) Activity Assay

Osteoblasts were induced with DEX, co-cultured with hUCMSCs, and treated with autophagy inhibitors in 6-well plates for 14 days. After the incubation period, cells were fixed with 4% paraformaldehyde for 15 min at room temperature, washed with PBS, and stained with the BCIP/NBT Alkaline Phosphatase Staining Kit (Beyotime, Shanghai, China). ALP activity was measured to evaluate osteoblast differentiation, and ALP activity was quantified using the Alkaline Phosphatase Assay Kit (Nanjing, China). The absorbance of ALP activity was measured at 520 nm using a Tecan Sunrise microplate reader.

### 2.8. Alizarin Red S Staining

Osteoblasts were induced with DEX, co-cultured with hUCMSCs, and treated with autophagy inhibitors in 6-well plates for 28 days. After the culture period, the excess medium was removed, and the cells were washed twice with PBS. Cells were then fixed with 4% paraformaldehyde for 15 min at room temperature, rinsed with PBS, and stained with 1 mL of Alizarin Red S (Solarbio, Beijing, China) for 30 min at room temperature. The wells were washed again with PBS to remove excess dye, and some PBS was retained at the bottom of the wells. Images were captured using a camera and microscope.

### 2.9. qRT-PCR

After osteoblast modeling, total RNA was extracted using Trizol reagent (A33254, Invitrogen, Waltham, MA, USA) according to the manufacturer’s instructions. The cDNA was synthesized using the First-Strand cDNA Synthesis Kit (AT301-02 Trans-Script First-Strand cDNA Synthesis Super-Mix, Trans-Gen Biotech, Beijing, China). Quantitative PCR (qPCR) was performed using the SYBR Green qPCR Kit (Takara, Shiga, Japan), with the following conditions: pre-denaturation at 95 °C for 5 min, denaturation at 95 °C for 30 s, annealing at 55 °C for 15 s, and extension at 72 °C for 30 s, with 39 cycles. Data were analyzed using the 2^−ΔΔCt^ method (Fluorescent Quantitative PCR Instruments; Thermo, Waltham, MA, USA). Three replicates were prepared for each sample group. Relative mRNA expression was normalized to the β-actin level. The following primer sequences were used: RUNX2: forward primer: 5′-ATCGCCTCAGTGATTTAGGG-3′; reverse primer: 5′-TGCCTGGGATCTGTAATCTG-3′ OPN: forward primer: 5′-GTGGGAAGGACAGTTATCAA-3′; reverse primer: 5′-GACTTTGGAAAGTTCCTG-3′. β-actin: forward primer: 5′-GTTGGAGCAAACATCCCCCA-3′; reverse primer: 5′-ACGCGACCATCCTCCTCTTA-3′.

### 2.10. Immunofluorescence Analysis

After the modeling was completed, the cells were washed twice with PBS, fixed in 4% paraformaldehyde pre-cooled at 4 °C for 30 min, then permeabilized with 0.2% Triton X-100 for 15 min, and incubated overnight at 4 °C with primary antibodies (LC3 (1:200; CST); LAMP2 (1:200; MBL, Nagoya, Japan)). The nuclei were then incubated with secondary antibody protected from light for 2 h. Finally, the nuclei were stained with Hoeschst for 5 min, and the stained cells and tissues were observed using an Olympus FV1000 confocal laser scanning microscope.

### 2.11. Western Blot Analysis

Total protein was extracted from each osteoblast group using RIPA lysis buffer (P0013B, Beyotime, Shanghai, China), and protein concentration was determined using the BCA kit (Servicebio, Wuhan, China). The protein (30–50 μg) was detached by 8–15% sodium dodecyl sulfate–polyacrylamide gel electrophoresis (SDS-PAGE) and transferred to the PVDF membrane. The membrane was blocked with 5% skimmed milk in PBS with 0.1% Tween 20 (PBST) buffer at 25 °C for 3 h. Proteins were separated by electrophoresis and transferred to membranes, which were incubated overnight at 4 °C with primary antibodies (OPN, RUNX2, LC3, PI3K, Beclin1, Bcl-2, Bax, caspase3; 1:1000 dilution, Cell Signaling Technology, USA; GAPDH from Servicebio, China). The membranes were subsequently incubated with secondary antibodies for 1 h at 37 °C. Protein levels were visualized and quantified using ImageJ software (v1.8.0.112).

### 2.12. Serological Testing

Blood was collected from the hearts of rabbits, centrifuged at 4 °C and 1500 r/min for 10 min, and the serum was separated. ALP activity in the serum was measured using the Alkaline Phosphatase (ALP) Activity Assay Kit (Nanjing Jianjian, China), following the manufacturer’s instructions.

### 2.13. Micro-CT Analysis

Femoral head tissue was analyzed using a MicroCT scanner (NEMO; NMC-200; Kunming, China) under optimized scanning conditions: *p* scanning voltage of 80 kV, scanning current of 0.06 mA, 20 μm layer thickness, AI of 1 mm, resolution of 2016 × 1344, and voxel size of 0.05 × 0.05 × 0.05 mm. After scanning, image reconstruction was performed using Recon software, and data acquisition was completed using Cruiser software. The target area was assessed for osteometric parameters, including trabecular spacing (Tb.Sp, mm), number of trabeculae (Tb.N, 1/mm), bone volume/total volume (BV/TV, %), and trabecular thickness (Tb.Th, mm).

### 2.14. Hematoxylin–Eosin (HE) Staining

Femoral head tissues were fixed in 10% paraformaldehyde overnight and then decalcified in 10% ethylenediaminetetraacetic acid for 2 months, and the decalcification solution was changed every 3 days. After decalcification, the specimens were dehydrated by an automatic dehydrator, embedded in paraffin, divided into sections of 5 μm thickness, soaked in xylene for 10 min to remove the paraffin, hydrated with gradient alcohol, stained with hematoxylin for 5 min, differentiated with 5% acetic acid for 5 min, stained with eosin for 3 min and dehydrated with gradient alcohol, and then placed under a microscope for observation and analyzed for the rate of bone cavitation in a high-magnification field of view.

### 2.15. Statistical Analysis

Biological specimens were collected and processed with strict adherence to protocols and all samples were statistically significant. Data are presented as mean ± S.E. Statistical analysis was performed using GraphPad Prism 8.0.2 software (the software was taken from https://www.graphpad.com/). Differences between groups were evaluated using one-way analysis of variance (ANOVA) with Bonferroni correction, and a *p*-value < 0.05 was considered statistically significant.

## 3. Results

### 3.1. Identification of hUCMSC Characteristics

**Figure 1:**
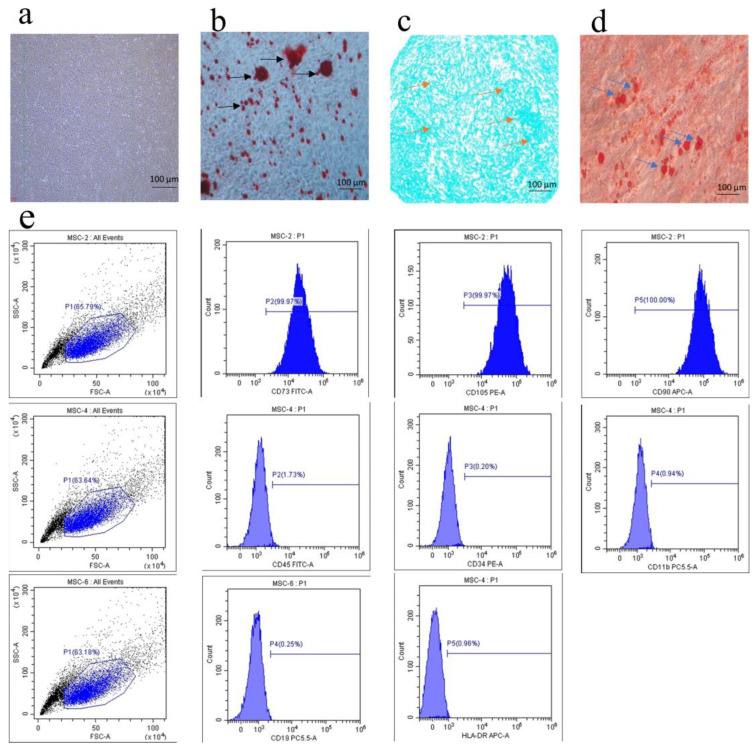
Characterization of human umbilical cord mesenchymal stem cells. (**a**) Optical microscopy revealed that hUCMSCs exhibited a typical spindle-shaped morphology (scale bar = 100 μm). (**b**–**d**) hUCMSCs demonstrated the ability to differentiate into various cell types under specific conditions: Black arrows indicate osteogenic mineralized nodules, orange arrows indicate chondrocytes, and blue arrows indicate adipocytes (scale bar = 100 μm). (**e**) Flow cytometry analysis of cell surface markers on hUCMSCs.

**Figure 2:**
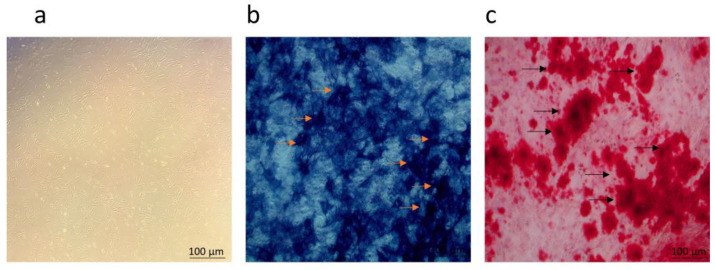
Characterization of osteoblasts. (**a**) Optical microscopy revealed that osteoblasts exhibited a typical spindle-shaped morphology (scale bar = 100 μm). (**b**) Positive alkaline phosphatase staining for osteoblasts, with yellow arrows indicating alkaline phosphatase (scale bar = 100 μm). (**c**) Positive Alizarin Red S staining for osteoblasts, with black arrows indicating osteoblastic mineralized nodules (scale bar = 100 μm).

hUCMSCs were cultured to the third generation, and their phenotype and multidirectional differentiation potential were assessed to confirm their identity as MSCs. Microscopic examination revealed that hUCMSCs exhibited typical spindle-shaped morphology and grew as a monolayer (Figure 1a). Under osteogenic, chondrogenic, and lipogenic induction cultures, hUCMSCs successfully differentiated into osteoblasts, chondrocytes, and adipocytes, as evidenced by the appearance of distinct cell types (Figure 1b–d). Flow cytometry analysis confirmed that the adherent cells expressed MSC markers CD73, CD105, and CD90, while lacking expression of hematopoietic markers CD45 and CD34, and immune markers HLA-DR, CD19, and CD11b, consistent with the characteristics of hUCMSCs reported in the literature [42] (Figure 1e). Osteoblasts were identified based on their osteogenic activity. Microscopic observation of active osteoblasts revealed a characteristic polygonal or pike-shaped morphology (Figure 2a). ALP staining and Alizarin Red S staining indicated the presence of alkaline phosphatase activity and the formation of mineralized nodules, further confirming the identity of the cells as osteoblasts (Figure 2b,c).

### 3.2. hUCMSCs Attenuated DEX-Induced Osteoblast Damage

**Figure 3:**
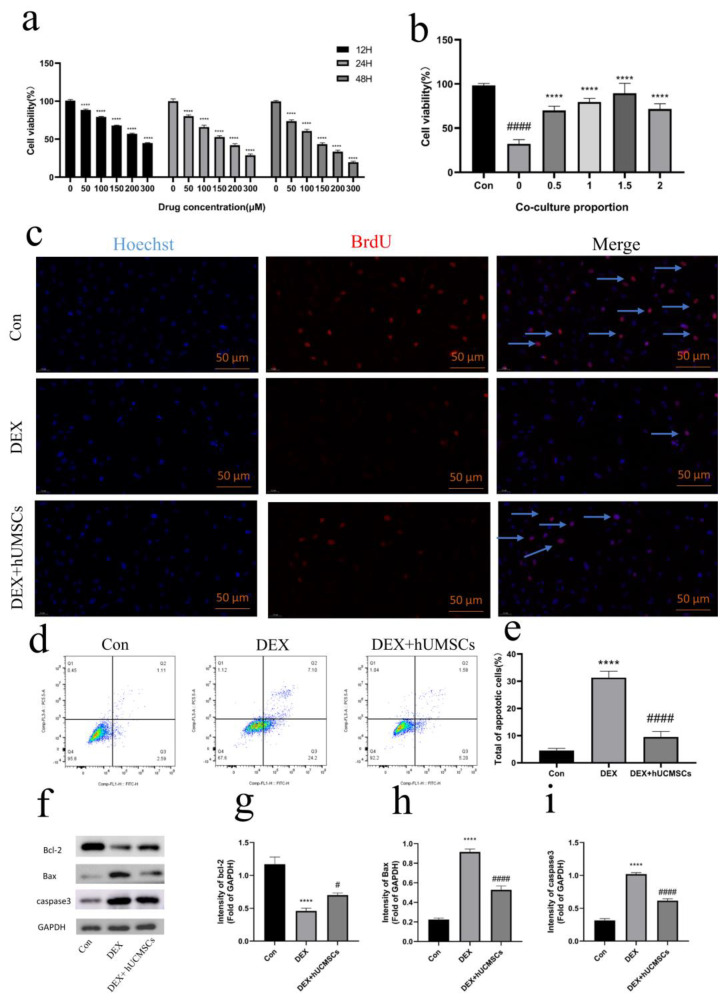
hUCMSCs attenuated DEX-induced osteoblast damage. (**a**) Osteoblasts were treated with various concentrations of DEX for different durations, and cell viability was measured by CCK8 assay. Values are presented as means ± S.E. (*n* = 5) from independent experiments. **** *p* < 0.0001, compared to the control group. (**b**) Osteoblasts were co-cultured with hUCMSCs at different ratios, and cell viability was measured by CCK8 assay. Values are presented as means ± S.E. (*n* = 5) from independent experiments. **** *p* < 0.0001, compared to the 0 group, and #### *p* < 0.0001, compared to the control group. (**c**) BrdU incorporation assay to assess the effect of 48 h of Dex treatment on osteoblast proliferation. Blue arrows indicate proliferating cells (scale bar = 50 μm). (**d**) Osteoblast apoptosis detected by flow cytometry following Annexin V-FITC and PI staining after 48 h of Dex treatment. (**e**) Quantitative analysis of the apoptotic rate from (**d**). (**f**) Western blot analysis of Bcl-2, Bax, and cleaved caspase-3 expression levels in osteoblasts after 48 h of Dex treatment. (**g**–**i**) Quantitative analysis of Western blot data from (**f**). Values are presented as means ± S.E. (*n* = 5) from independent experiments. **** *p* < 0.0001 compared to the control group, and #### *p* < 0.0001, # *p* < 0.05 compared to the DEX group.

DEX induces significant damage to osteoblasts by impairing both cell survival and function. To assess whether hUCMSCs could mitigate DEX-induced damage, osteoblasts were exposed to varying concentrations of DEX for specific durations to identify the optimal treatment conditions. The CCK8 assay demonstrated a dose-dependent cytotoxicity of DEX on osteoblasts, with both 150 μM and 200 μM DEX treatments for 48 h significantly reducing cell viability compared to the control group (*p* < 0.0001) (Figure 3a). Specifically, 200 μM DEX treatment for 48 h resulted in a 31.26% reduction in cell viability compared to the control group, establishing this concentration as the baseline for subsequent experiments. In the next step, osteoblasts were co-cultured with hUCMSCs at different cell densities (0.5, 1, 1.5, 2) for 12 h, followed by continued DEX induction for 48 h. The CCK8 assay revealed that hUCMSC co-culture significantly enhanced osteoblast viability (*p* < 0.0001), with the maximal protective effect observed at a 1.5:1 cell ratio (Figure 3b). This ratio was used for all subsequent co-culture experiments. To further evaluate the effects on osteoblast proliferation, BrdU assays showed that DEX treatment significantly inhibited cell proliferation, whereas hUCMSC co-culture substantially counteracted this inhibition (Figure 3c). Additionally, Western blot analysis indicated upregulation of cleaved caspase 3 in DEX-treated osteoblasts (*p* < 0.0001), and Annexin V/PI staining combined with flow cytometry revealed a marked increase in apoptosis (*p* < 0.0001) (Figure 3d–i). In contrast, hUCMSC co-culture notably reduced Bax expression (*p* < 0.0001) and significantly decreased apoptosis incidence (*p* < 0.0001) (Figure 3d–i), suggesting that hUCMSCs effectively mitigate DEX-induced apoptosis.

### 3.3. hUCMSCs Ameliorated DEX-Induced Inhibition of Osteogenesis

**Figure 4:**
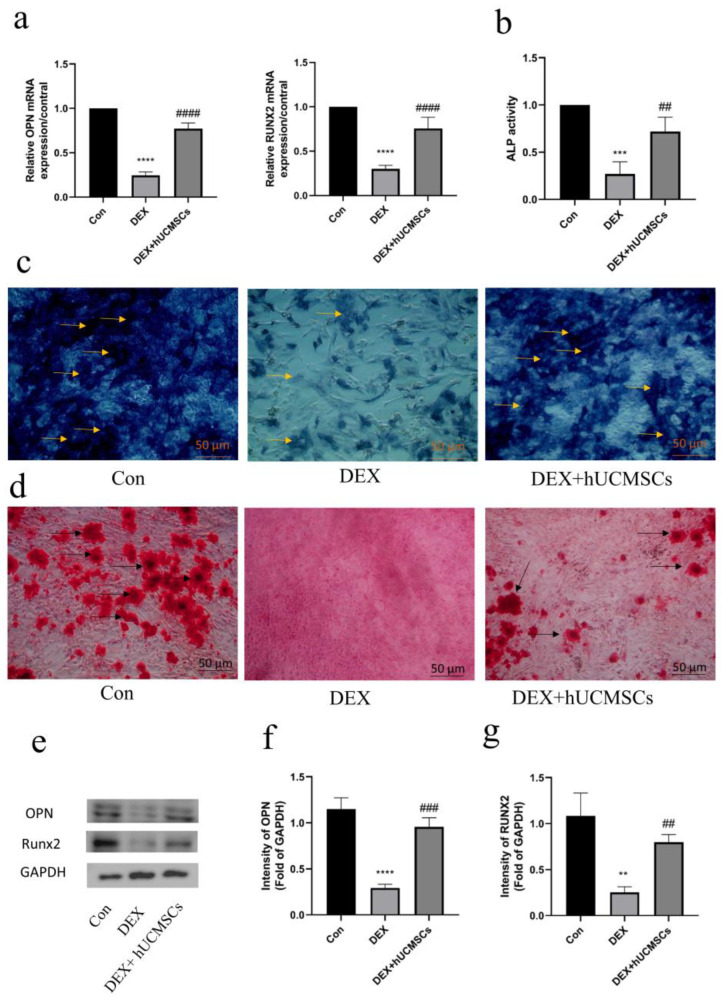
hUCMSCs ameliorated DEX-induced inhibition of osteogenesis. (**a**) mRNA-relative expression levels of RUNX2 and OPN detected by qPCR. (**b**) Alkaline phosphatase (ALP) activity measured with an ALP assay kit. (**c**) Alkaline phosphatase staining showing that co-culture with hUCMSCs attenuated the Dex-induced decrease in ALP activity in osteoblasts. Yellow arrows indicate alkaline phosphatase (scale bar = 50 μm). (**d**) Alizarin Red S staining showing that co-culture with hUCMSCs attenuated the Dex-induced decrease in osteoblast mineralization. Black arrows indicate osteoblastic mineralized nodules (scale bar = 50 μm). (**e**–**g**) Western blot analysis of OPN and RUNX2 expression levels in osteoblasts following 48 h of Dex treatment. Values are presented as means ± S.E. (*n* = 5) from independent experiments. **** *p* < 0.0001, *** *p* < 0.001, ** *p* < 0.01 compared to the control group, and #### *p* < 0.0001, ### *p* < 0.001, ## *p* < 0.01, compared to the DEX group.

The protective effect of hUCMSCs against DEX-induced inhibition of osteoblast differentiation was further investigated. Real-time PCR analysis revealed that hUCMSCs significantly mitigated the DEX-induced downregulation of OPN and Runx2 mRNA expression in osteoblasts (*p* < 0.0001) (Figure 4a). Additionally, ALP activity, a key marker of osteoblast differentiation and maturation, was assessed through ALP staining and enzyme activity assays. The results demonstrate that hUCMSC co-culture counteracted the DEX-induced reduction in ALP activity, as confirmed by both alkaline phosphatase assays and staining (*p* < 0.01) (Figure 4b,c). Furthermore, Alizarin Red S staining was performed to evaluate osteoblast mineralization. After 28 days of DEX treatment, with or without hUCMSC co-culture, the results reveal that hUCMSCs significantly alleviated DEX-induced inhibition of mineralization in osteoblasts (Figure 4d).

Western blot analysis was conducted to assess the expression levels of osteogenesis-related proteins, osteopontin (OPN), and the osteogenic transcription factor Runx2 in osteoblasts. The results demonstrate that DEX treatment led to a significant downregulation of both OPN and Runx2 in osteoblasts (*p* < 0.0001). However, co-culture with hUCMSCs effectively mitigated these reductions (*p* < 0.01) (Figure 4e–g), indicating that hUCMSCs alleviate DEX-induced osteogenic inhibition in osteoblasts, thereby promoting osteogenesis.

### 3.4. hUCMSCs Enhanced Autophagy in DEX-Induced Osteoblasts

**Figure 5:**
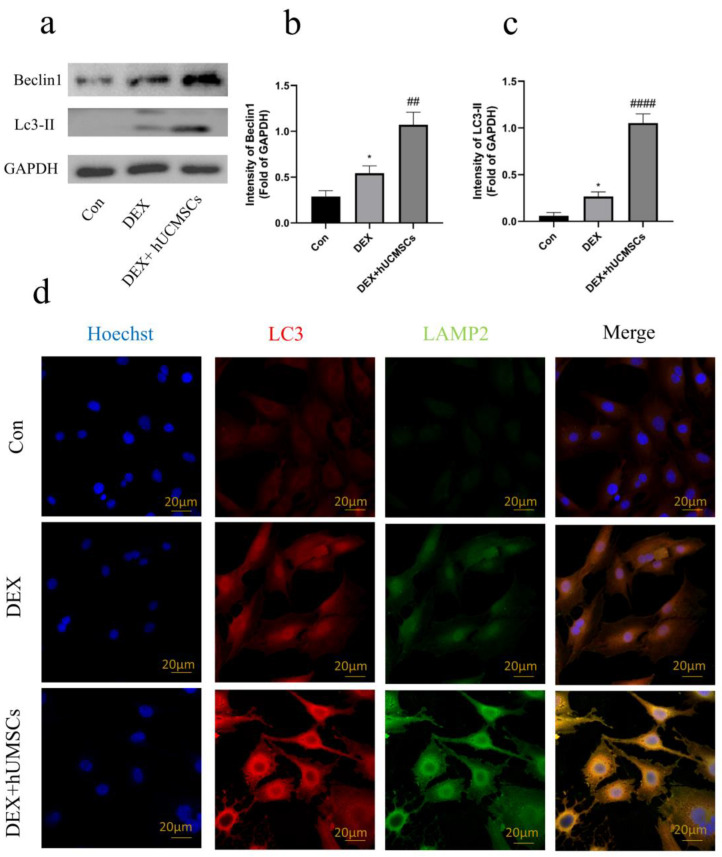
hUCMSCs enhanced autophagy in DEX-induced osteoblasts. (**a**) Western blot analysis of Beclin1 and LC3-II expression levels in osteoblasts following 48 h of Dex treatment. (**b**,**c**) Quantitative analysis of Western blot data from (**a**). (**d**) Immunofluorescence analysis to assess the number of LC3-positive (red) autophagosomes colocalized with LAMP2-labeled (green) lysosomes (scale bar = 20 μm). Values are presented as means ± S.E. (*n* = 5) from independent experiments. * *p* < 0.05 compared to the control group, an #### *p* < 0.0001, ## *p* < 0.01compared to the DEX group.

Extensive evidence highlights the protective role of autophagy in various disease states. To investigate whether hUCMSCs exert a protective effect through modulation of autophagy in osteoblasts under DEX treatment, the expression of key autophagy-related proteins was assessed. Western blot analysis revealed that Beclin1 and LC3-II expression were significantly elevated in the DEX-treated group compared to the control group (*p* < 0.05). Interestingly, hUCMSC co-culture further enhanced the expression levels of Beclin1 and LC3-II (*p* < 0.01), suggesting that hUCMSCs promote autophagy in osteoblasts under DEX conditions (Figure 5a–c).

However, an increase in autophagy-related proteins alone does not fully explain the autophagic process, as autophagy requires the fusion of autophagosomes with lysosomes to form autophagic lysosomes capable of degradation. To determine whether hUCMSCs promote this process, immunofluorescence analysis was performed. The results indicate that, while the DEX group displayed a higher number of autophagosomes, fusion with lysosomes was limited. In contrast, hUCMSCs significantly enhanced the formation of autophagic lysosomes (Figure 5d), supporting the notion that hUCMSCs promote autophagolysosome formation.

Overall, although DEX-treated osteoblasts exhibited some increase in autophagy, the absence of fusion between autophagosomes and lysosomes limited the protective potential of the autophagic process. In contrast, co-culture with hUCMSCs facilitated the fusion of autophagosomes with lysosomes, thereby enhancing the autophagic response and enabling a protective effect on osteoblasts.

### 3.5. Inhibition of Autophagy Attenuated the Protective Effect of hUCMSCs on DEX-Induced Osteoblasts

**Figure 6:**
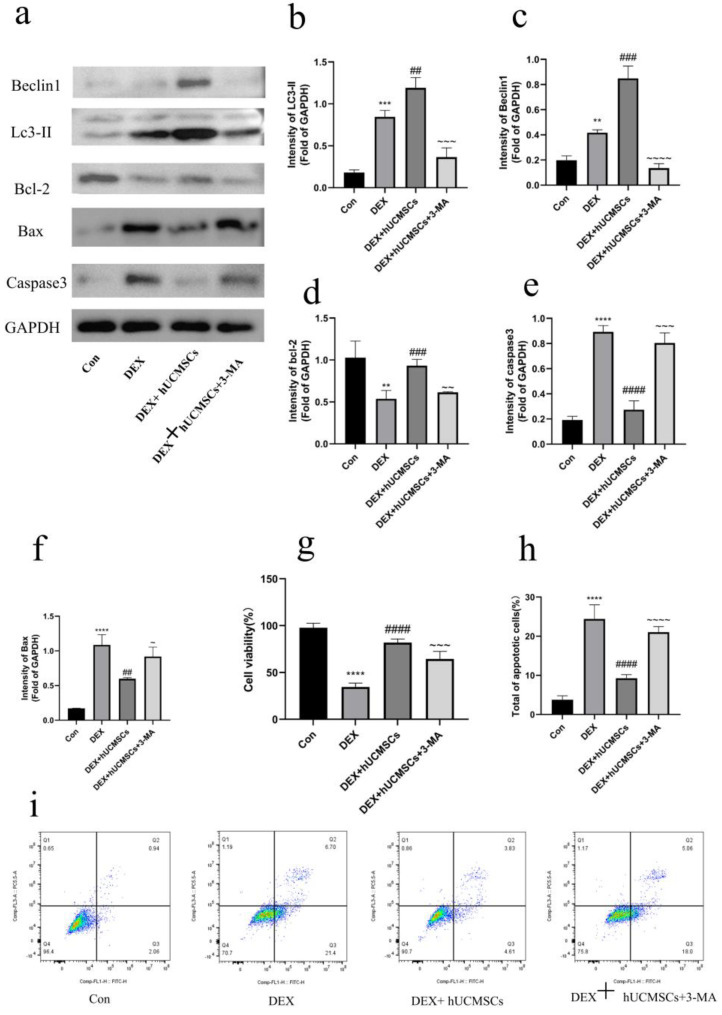
Inhibition of autophagy attenuated the protective effect of hUCMSCs on DEX-induced osteoblasts. (**a**) Western blot analysis of Beclin1, LC3-II, Bax, Bcl-2, and caspase3 expression levels in osteoblasts following 48 h of Dex treatment. (**b**−**f**) Quantitative analysis of Western blot data from (**a**). (**g**) 3-MA significantly decreased cell viability. (**h**) Quantitative analysis of apoptotic rate shown in (**i**). (**i**) Inhibition of autophagy with 3-MA significantly increased the number of apoptotic cells. Values are presented as means ± S.E. (*n* = 5) from independent experiments. **** *p* < 0.0001, *** *p* < 0.001, ** *p* < 0.01 compared to the control group. #### *p* < 0.0001, ### *p* < 0.001, ## *p* < 0.01, compared to the DEX group. ~ *p* < 0.05, ~~ *p* < 0.01, ~~~ *p* < 0.001, ~~~~ *p* < 0.0001, compared to the DEX + hUCMSC group.

**Figure 7:**
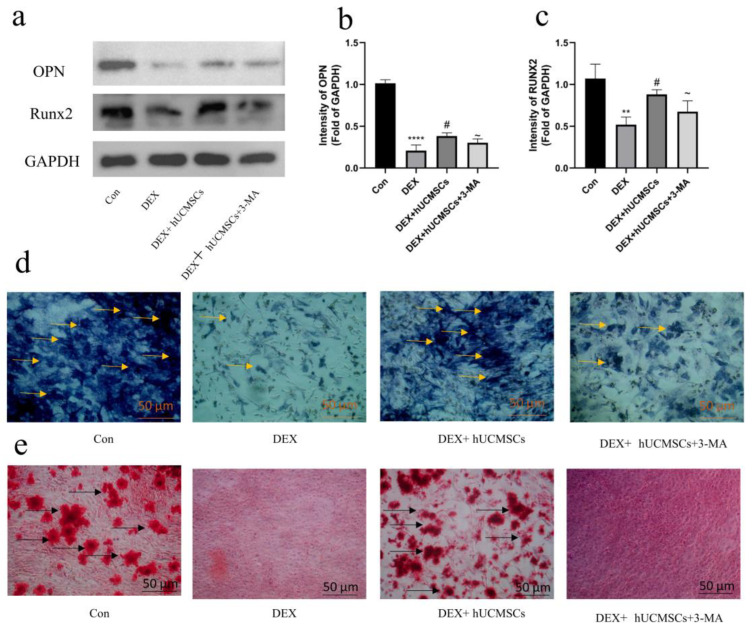
Inhibition of autophagy affected the osteoprotective effect of hUCMSCs on osteoblasts. (**a**) Western blot analysis of OPN and RUNX2 expression levels in osteoblasts after 48 h of Dex treatment. (**b**,**c**) Quantitative analysis of Western blot data in (**a**). (**d**) Alkaline phosphatase staining showing that inhibition of autophagy diminishes the osteoprotective effect of hUCMSCs on osteoblasts. Yellow arrows indicate alkaline phosphatase activity (scale bar = 50 μm). (**e**) Alizarin red S staining indicating that autophagy inhibition impacts the osteoprotective effect of hUCMSCs on osteoblasts. Black arrows indicate osteoblastic mineralized nodules (scale bar = 50 μm). Values are expressed as means ± S.E. (*n* = 5) from independent experiments. **** *p* < 0.0001, ** *p* < 0.01 compared to the control group. # *p* < 0.05 compared to the DEX group. ~ *p* < 0.05, compared to the DEX + hUCMSC group.

Previous findings have demonstrated that hUCMSC co-culture enhances autophagy in osteoblasts, protecting them from DEX-induced apoptosis and osteogenic inhibition. To further investigate whether this protective effect is mediated through the promotion of autophagy, 3-MA, an autophagy inhibitor, was introduced to the medium to suppress the autophagic process in osteoblasts. As shown in Figure 6a–c, treatment with 3-MA significantly reduced the expression of autophagy-related proteins in osteoblasts co-cultured with hUCMSCs (*p* < 0.001), confirming the inhibitory effect of 3-MA on autophagy and validating that hUCMSCs promote autophagic activity in DEX-treated osteoblasts. Moreover, inhibition of autophagy by 3-MA notably decreased cell viability (*p* < 0.001) (Figure 6g), upregulated the expression of apoptosis-related proteins (*p* < 0.05) (Figure 6a,d–f), and increased the number of apoptotic cells relative to the hUCMSC co-culture group (*p* < 0.0001) (Figure 6h,i). Importantly, the expression levels of bone-formation markers were significantly lower in the 3-MA-treated group compared to the hUCMSC co-culture group (*p* < 0.05) (Figure 7a–c), suggesting that autophagy inhibition impaired the osteoprotective effects of hUCMSCs on osteoblasts. This was further confirmed by ALP staining and Alizarin Red S staining, which indicated reduced osteogenic activity in the 3-MA-treated group (Figure 7d,e).

Overall, the inhibition of autophagy compromised the protective effects of hUCMSCs on osteoblast function and survival under DEX-induced conditions. These results suggest that hUCMSCs mitigate DEX-induced osteoblast damage by modulating autophagy.

### 3.6. hUCMSC Infusion Improved the Femoral Head Microstructure in the MPS-Induced ONFH Rabbit Model

**Figure 8:**
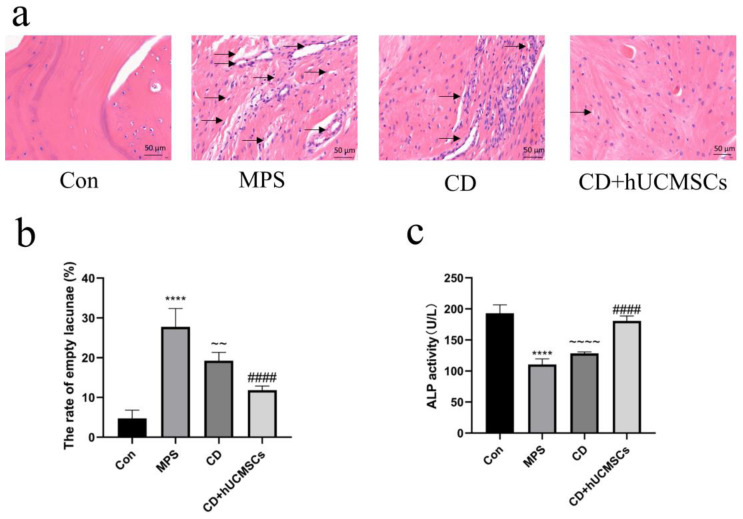
hUCMSC infusion improved the femoral head microstructure in the Mps-induced ONFH rabbit model. (**a**) HE staining of rabbit femoral head tissue, with black arrows indicating empty lacunae (scale bar = 50 μm). (**b**) Quantitative analysis of the percentage of empty lacunae, as shown in (**a**). (**c**) Measurement of ALP activity in rabbit serum. Data are expressed as means ± S.E. (*n* = 12). **** *p* < 0.0001, compared to the Con group. #### *p* < 0.0001, compared to the MPS group. ~~ *p* < 0.01, ~~~~ *p* < 0.0001, compared to the CD + hUCMSC group.

Following modeling, serum ALP activity was assessed in rabbits from the four groups. The ALP activity in the CD + hUCMSC group was significantly higher compared to the MPS group (*p* < 0.0001) (Figure 8c). HE staining results reveal that the femoral head of the Con group exhibited normal histological structure, with tightly arranged bone trabeculae, uniform staining of osteoblast cytoplasm and nuclei, and dense osteoclasts. In contrast, the MPS group showed disrupted trabeculae, sparse arrangement, reduced osteoblast numbers, increased bone fossae, and shrunken nuclei. The CD group showed some improvement in trabecular condition, but bone fossae and reduced osteoblast numbers remained. Notably, the trabecular structure in the CD + hUCMSC group was significantly improved, with increased osteoblasts, denser osteocytes, and fewer bone fossae and shrunken osteoblast nuclei (*p* < 0.0001) (Figure 8a,b). These results suggest that hUCMSC infusion significantly improves femoral bone microarchitecture and effectively reverses ONFH in the GC-induced rabbit ONFH model.

**Figure 9:**
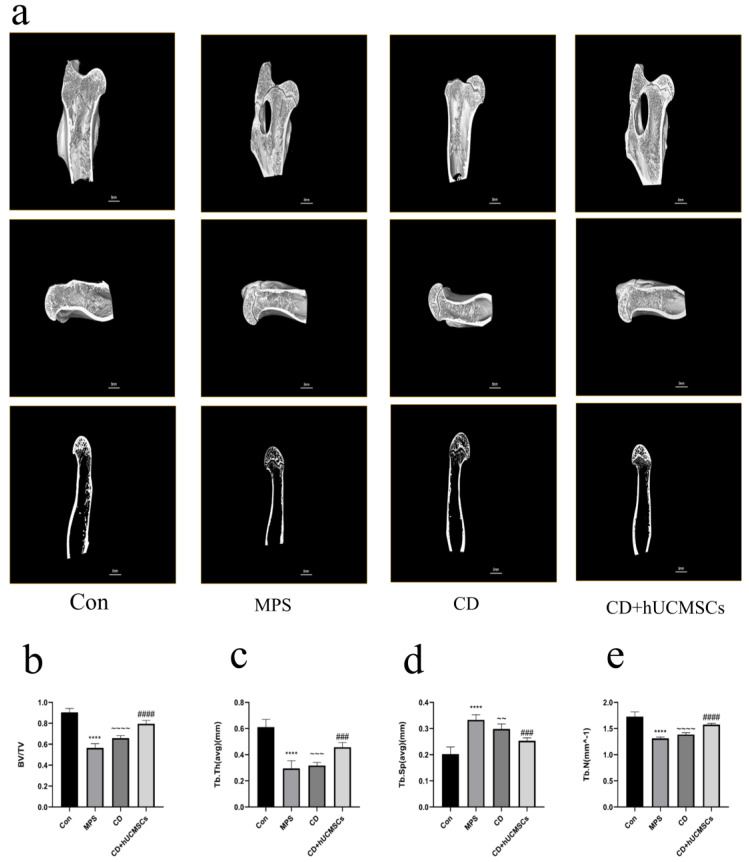
hUCMSC infusion improved the femoral head microstructure in the MPS-induced ONFH rabbit model. (**a**) Representative images of Micro-CT of the femoral head in each group. (**b**–**e**) Quantitative analysis of Tb.Th, Tb.N, BV/TV, and Tb.Sp in each group. Data are presented as means ± S.E. (*n* = 12). **** *p* < 0.0001, compared to the Con group. ### *p* < 0.001, #### *p* < 0.0001 compared to the MPS group. ~~ *p* < 0.01, ~~~ *p* < 0.001, ~~~~ *p* < 0.0001, compared to the CD + hUCMSC group.

Micro-CT was employed to quantitatively analyze the microstructure of the femoral head, with the results presented in Figure 9a. In the Con group, the bone trabeculae were intact and tightly arranged, while in the MPS group, the trabeculae were sparse and disorganized. The trabecular structure in the CD + hUCMSC group showed significant improvement. The Tb.Th, Tb.N, and BV/TV values were notably higher in the CD + hUCMSC group compared to the MPS group (*p* < 0.0001), while the Tb.Sp values were significantly lower (*p* < 0.0001) (Figure 9b–e). These results align with the histological observations from HE staining and further support that hUCMSC infusion effectively inhibits the progression of ONFH in rabbits, promotes bone regeneration, and improves the microstructural integrity of the femoral head.

## 4. Discussion

This study is the first to validate the therapeutic effect of hUCMSCs in an in vivo model of rabbit GC-ONFH, demonstrating that hUCMSC infusion can significantly improve the microstructure of the femoral head and promote bone regeneration. To better understand the underlying mechanisms by which hUCMSCs influence bone metabolism, in vitro experiments were conducted using a DEX-stimulated osteoblast model. Our findings indicate that hUCMSCs delay the progression of GC-ONFH by inhibiting osteoblast apoptosis and promoting osteogenesis. Furthermore, hUCMSCs exert therapeutic effects by modulating osteoblast autophagy. Taken together, the results from both in vivo and in vitro studies support the hypothesis that hUCMSCs protect osteoblasts from GC-induced injury through the regulation of autophagy.

The widespread clinical use of hormonal drugs has led to an increase in the number of cases of GC-induced ONFH. Clinical studies have shown that hormones are the primary cause of ONFH [43], accounting for more than 57% of all cases [44]. Long-term, high-dose GC use can lead to femoral head necrosis, with the incidence of ONFH rising significantly when the cumulative prednisone dose exceeds 200 mg [45]. GC-induced ONFH damages femoral blood vessels and suppresses osteogenic activity, ultimately impairing bone metabolism [46]. Bone homeostasis relies on a delicate balance between bone formation and resorption, driven by the interaction of osteoblasts and osteoclasts. However, high doses of GC severely inhibit osteoblast function, leading to an imbalance in this homeostasis [47]. Additionally, GC treatment suppresses the expression of osteogenic genes, causing a decrease in osteoblast numbers, which leads to osteoporosis, damage to the bone support structures, collapse of the femoral head, and, ultimately, ONFH [48]. In the present study, the rabbit GC-ONFH model exhibited increased serum ALP activity, impaired femoral head microstructure, and disorganized trabecular structure, all of which confirmed the onset of femoral head necrosis. In parallel, DEX-treated osteoblasts showed increased apoptosis and reduced osteogenic activity, further demonstrating that prolonged GC use negatively impacts osteoblast function and contributes to femoral head necrosis. Therefore, identifying drugs or mechanisms that can protect osteoblasts from GC-induced damage has become a critical issue in the clinical management of ONFH.

The present study further demonstrated that hUCMSC infusion mitigated the DEX-induced increase in osteoblast apoptosis and attenuated the reduction in osteogenic activity. Additionally, in vitro experiments revealed that hUCMSC infusion significantly improved bone microarchitecture. Accumulating evidence supports the capacity of MSCs to not only repair and regenerate damaged tissues but also modulate immune responses, offering substantial therapeutic potential in treating various conditions, including neurological disorders, type 2 diabetes, and hematopoietic disorders [49]. The clinical feasibility of MSC transplantation has been well established. For instance, Peng et al. reported that hUCMSC-derived extracellular vesicles (hUCMSC-EVs) exerted a protective effect in rat SANFH by activating the PI3K/AKT signaling pathway, thereby inhibiting osteoblast apoptosis and preventing disease progression [42]. Similarly, Yoon et al. demonstrated that cultured autologous adipose-derived MSCs effectively treated femoral head necrosis [50]. Furthermore, Yang et al. demonstrated that both hUCB-MSCs and rBM-MSCs could repair cartilage damage to a certain extent [51]. These observations align with the results of the current study, which showed that hUCMSC infusion inhibited osteoblast apoptosis and delayed the progression of GC-induced ONFH.

Stem cell therapy faces significant challenges due to the harsh microenvironment at the lesion site following local injection, which affects the survival and functionality of transplanted cells. Research indicates that less than 5% of the transplanted cells survive at the injection site within hours after transplantation [52]. To enhance the viability and therapeutic efficacy of hUCMSCs, CD was combined with hUCMSC infusion. To assess the specific impact of hUCMSC infusion, a CD-only group was included to control for any potential confounding effects of CD. CD is a well-established surgical procedure for treating early-stage ONFH, particularly in patients with femoral head collapse [53]. However, in clinical practice, the success rate of CD alone remains suboptimal due to challenges such as insufficient bone regeneration, imprecise localization, incomplete necrotic tissue removal, limited indications, and the risk of fractures [54]. As artificial joint surgery has advanced, hip replacement has become the primary treatment for middle- and late-stage femoral head necrosis. However, the risk of failure in early-stage femoral head necrosis remains high, as these patients tend to be younger, more active, and more likely to seek hip preservation therapies. Therefore, improving medullary CD remains a major area of research. To achieve better clinical outcomes, the combination of MSC therapy with CD has emerged as a promising strategy. MSCs possess the ability to repair necrotic bone tissue and can function as repair precursor cells, secreting various cytokines and growth factors that initiate the healing process within the femoral head [55]. Moreover, hUCMSCs offer advantages such as low immunogenicity, easy availability, and minimal ethical concerns, making them an ideal candidate for cell-based therapies. In our in vivo experiments, hUCMSC infusion improved the microstructure of the rabbit femoral head and mitigated the development of osteonecrosis in the hormonal ONFH model. These observations suggest that hUCMSCs may prevent the onset of hormonal ONFH by enhancing bone microstructure. This study further confirms the significant therapeutic efficacy of hUCMSC infusion in early-stage femoral head osteonecrosis. The combination of CD and hUCMSCs presents a promising therapeutic approach and holds substantial clinical relevance for the treatment of early-stage osteonecrosis of the femoral head.

This study provides the first evidence that hUCMSCs exert their therapeutic effect by modulating autophagy in osteoblasts. In particular, a significant elevation of the autophagy-related proteins LC3 and Beclin1 was observed in the DEX + hUCMSC group compared to the control (Con) and DEX groups. This increase in autophagy was accompanied by a reduction in osteoblast apoptosis and an enhancement in osteogenic activity in the DEX + hUCMSC group. However, when the autophagy inhibitor 3-MA was applied, the therapeutic effect of hUCMSCs was markedly diminished, further supporting the critical role of autophagy in mediating its protective effects. Extensive evidence underscores the importance of autophagy in inhibiting apoptosis across various disease states. For instance, Jiao et al. demonstrated that upregulation of autophagy and the formation of autophagic lysosomes facilitated the clearance of α-synuclein aggregates, thereby preventing dopaminergic neuron degeneration in Parkinson’s disease and improving the associated pathological changes [56]. Similarly, Fan et al. showed that autophagy regulates the inflammatory signaling pathway in acute epithelial cell (AEC) injuries and pulmonary ischemia–reperfusion injuries, with rapamycin acting as an autophagy promoter to attenuate lung inflammation caused by these injuries [57]. Moreover, Han et al. found that while autophagy was induced in β-cells under chronic high glucose conditions, this mild increase was insufficient to prevent β-cell dysfunction and cellular impairment caused by high glucose exposure [58]. This finding is consistent with our observation that, while autophagosome formation was slightly increased in DEX-treated osteoblasts, there was limited fusion between autophagosomes and lysosomes. Notably, several studies have highlighted the capacity of MSCs to enhance autophagy. For example, Song et al. reported that MSCs improve diabetes-induced muscular dystrophy by enhancing AMPK/ULK1-mediated autophagy through exocytosis [59]. In addition, MSC therapy has been shown to promote neuronal survival in Alzheimer’s disease and Parkinson’s disease models by augmenting autophagic activity [60]. In line with these observations, our study demonstrates that hUCMSC infusion can enhance both autophagosome and autophagic lysosome formation, thereby exerting cytoprotective effects on osteoblasts.

Despite the strengths of this study, there are several limitations that should be acknowledged. While the data suggest that hUCMSCs exert a therapeutic effect by modulating autophagy, the current study primarily focuses on phenotypic observations. Therefore, the precise molecular mechanisms underlying the regulation of cellular autophagy by hUCMSCs remain to be elucidated. Additionally, the in vivo role of hUCMSCs is multifaceted, as they may also participate in immunomodulation through the secretion of cytokines and non-coding RNAs, which could contribute to their therapeutic effects. Furthermore, hUCMSCs may exert protective roles on other cell types, such as vascular endothelial cells and immune cells, which warrants further investigation and constitutes a focus of our ongoing research. Another important consideration is that large-scale clinical studies are crucial to assessing the safety, long-term efficacy, and optimal dosing strategies for hUCMSCs as an adjunctive therapy in the early treatment of GC-ONFH.

In conclusion, this study demonstrates that hUCMSCs enhance autophagy and protect osteoblasts from DEX-induced injury and osteogenesis inhibition in vitro. Moreover, hUCMSC infusion significantly reduced the incidence of GC-ONFH in a rabbit model. Taken together, these findings suggest that hUCMSCs exert a protective effect on osteoblasts under GC conditions by modulating autophagy. This study provides new insights into the therapeutic mechanisms of stem cell therapy for GC-ONFH and lays a critical foundation for the future clinical application of MSCs in the treatment of this condition.

## Data Availability

All data are presented in the manuscript.

## References

[1] Zhang S., Wang C., Shi L., Xue Q. (2021). Beware of Steroid-Induced Avascular Necrosis of the Femoral Head in the Treatment of COVID-19-Experience and Lessons from the SARS Epidemic. Drug Des. Dev. Ther..

[2] Chen K., Liu Y., He J., Pavlos N., Wang C., Kenny J., Yuan J., Zhang Q., Xu J., He W. (2020). Steroid-induced osteonecrosis of the femoral head reveals enhanced reactive oxygen species and hyperactive osteoclasts. Int. J. Biol. Sci..

[3] Wang W., Jiang H., Yu J., Lou C., Lin J. (2024). Astaxanthin-mediated Nrf2 activation ameliorates glucocorticoid-induced oxidative stress and mitochondrial dysfunction and impaired bone formation of glucocorticoid-induced osteonecrosis of the femoral head in rats. J. Orthop. Surg. Res..

[4] Chang C., Greenspan A., Gershwin M.E. (2020). The pathogenesis, diagnosis and clinical manifestations of steroid-induced osteonecrosis. J. Autoimmun..

[5] Zhang J., Cao J., Liu Y., Zhao H. (2024). Advances in the Pathogenesis of Steroid-Associated Osteonecrosis of the Femoral Head. Biomolecules.

[6] Konarski W., Poboży T., Konarska K., Śliwczyński A., Kotela I., Hordowicz M., Krakowiak J. (2023). Osteonecrosis Related to Steroid and Alcohol Use-An Update on Pathogenesis. Healthcare.

[7] Liu Y., Shan H., Zong Y., Lin Y., Xia W., Wang N., Zhou L., Gao Y., Ma X., Jiang C. (2021). IKKe in osteoclast inhibits the progression of methylprednisolone-induced osteonecrosis. Int. J. Biol. Sci..

[8] Kong N., Yang H., Tian R., Liu G., Li Y., Guan H., Wei Q., Du X., Lei Y., Li Z. (2022). An injectable self-adaptive polymer as a drug carrier for the treatment of nontraumatic early-stage osteonecrosis of the femoral head. Bone Res..

[9] Lin T., Kohno Y., Huang J.F., Romero-Lopez M., Maruyama M., Ueno M., Pajarinen J., Nathan K., Yao Z., Yang F. (2019). Preconditioned or IL4-Secreting Mesenchymal Stem Cells Enhanced Osteogenesis at Different Stages. Tissue Eng. Part A.

[10] Li J., Yan S., Han W., Dong Z., Li J., Wu Q., Fu X. (2022). Phospholipid-grafted PLLA electrospun micro/nanofibers immobilized with small extracellular vesicles from rat adipose mesenchymal stem cells promote wound healing in diabetic rats. Regen. Biomater..

[11] Tan Y., Huang Y., Mei R., Mao F., Yang D., Liu J., Xu W., Qian H., Yan Y. (2022). HucMSC-derived exosomes delivered BECN1 induces ferroptosis of hepatic stellate cells via regulating the xCT/GPX4 axis. Cell Death Dis..

[12] Sun D.Z., Abelson B., Babbar P., Damaser M.S. (2019). Harnessing the mesenchymal stem cell secretome for regenerative urology. Nat. Rev. Urol..

[13] Li K., Yan G., Huang H., Zheng M., Ma K., Cui X., Lu D., Zheng L., Zhu B., Cheng J. (2022). Anti-inflammatory and immunomodulatory effects of the extracellular vesicles derived from human umbilical cord mesenchymal stem cells on osteoarthritis via M2 macrophages. J. Nanobiotechnol..

[14] Yao G., Qi J., Liang J., Shi B., Chen W., Li W., Tang X., Wang D., Lu L., Chen W. (2019). Mesenchymal stem cell transplantation alleviates experimental Sjögren’s syndrome through IFN-β/IL-27 signaling axis. Theranostics.

[15] Zhou H., He Y., Xiong W., Jing S., Duan X., Huang Z., Nahal G.S., Peng Y., Li M., Zhu Y. (2023). MSC based gene delivery methods and strategies improve the therapeutic efficacy of neurological diseases. Bioact. Mater..

[16] Jin L., Sun Z., Liu H., Zhu X., Zhou Y., Fu B., Zheng X., Song K., Tang B., Wu Y. (2021). Inflammatory monocytes promote pre-engraftment syndrome and tocilizumab can therapeutically limit pathology in patients. Nat. Commun..

[17] Jipa A., Vedelek V., Merényi Z., Ürmösi A., Takáts S., Kovács A.L., Horváth G.V., Sinka R., Juhász G. (2021). Analysis of Drosophila Atg8 proteins reveals multiple lipidation-independent roles. Autophagy.

[18] Taucher E., Mykoliuk I., Fediuk M., Smolle-Juettner F.M. (2022). Autophagy, Oxidative Stress and Cancer Development. Cancers.

[19] Teramoto K., Tsurekawa Y., Suico M.A., Kaseda S., Omachi K., Yokota T., Kamura M., Piruzyan M., Kondo T., Shuto T. (2020). Mild electrical stimulation with heat shock attenuates renal pathology in adriamycin-induced nephrotic syndrome mouse model. Sci. Rep..

[20] Prasher P., Sharma M., Singh S.K., Gulati M., Chellappan D.K., Zacconi F., De Rubis G., Gupta G., Sharifi-Rad J., Cho W.C. (2022). Luteolin: A flavonoid with a multifaceted anticancer potential. Cancer Cell Int..

[21] Luo P., Gao F., Han J., Sun W., Li Z. (2018). The role of autophagy in steroid necrosis of the femoral head: A comprehensive research review. Int. Orthop..

[22] Jing X., Yang F., Shao C., Wei K., Xie M., Shen H., Shu Y. (2019). Role of hypoxia in cancer therapy by regulating the tumor microenvironment. Mol. Cancer.

[23] Bai S.C., Xu Q., Li H., Qin Y.F., Song L.C., Wang C.G., Cui W.H., Zheng Z., Yan D.W., Li Z.J. (2019). NADPH Oxidase Isoforms Are Involved in Glucocorticoid-Induced Preosteoblast Apoptosis. Oxid. Med. Cell Longev..

[24] Tang Y.H., Yue Z.S., Li G.S., Zeng L.R., Xin D.W., Hu Z.Q., Xu C.D. (2018). Effect of β-ecdysterone on glucocorticoid-induced apoptosis and autophagy in osteoblasts. Mol. Med. Rep..

[25] Harr M.W., McColl K.S., Zhong F., Molitoris J.K., Distelhorst C.W. (2010). Glucocorticoids downregulate Fyn and inhibit IP(3)-mediated calcium signaling to promote autophagy in T lymphocytes. Autophagy.

[26] Li G., Song Y., Liao Z., Wang K., Luo R., Lu S., Zhao K., Feng X., Liang H., Ma L. (2020). Bone-derived mesenchymal stem cells alleviate compression-induced apoptosis of nucleus pulposus cells by N6 methyladenosine of autophagy. Cell Death Dis..

[27] Chen C.Y., Rao S.S., Yue T., Tan Y.J., Yin H., Chen L.J., Luo M.J., Wang Z., Wang Y.Y., Hong C.G. (2022). Glucocorticoid-induced loss of beneficial gut bacterial extracellular vesicles is associated with the pathogenesis of osteonecrosis. Sci. Adv..

[28] Liang X.Z., Luo D., Chen Y.R., Li J.C., Yan B.Z., Guo Y.B., Wen M.T., Xu B., Li G. (2022). Identification of potential autophagy-related genes in steroid-induced osteonecrosis of the femoral head via bioinformatics analysis and experimental verification. J. Orthop. Surg. Res..

[29] Yang Y., Klionsky D.J. (2020). Autophagy and disease: Unanswered questions. Cell Death Differ..

[30] Cen S., Wang P., Xie Z., Yang R., Li J., Liu Z., Wang S., Wu X., Liu W., Li M. (2019). Autophagy enhances mesenchymal stem cell-mediated CD4(+) T cell migration and differentiation through CXCL8 and TGF-β1. Stem Cell Res. Ther..

[31] Han H., Chen M., Li Z., Zhou S., Wu Y., Wei J. (2022). Corosolic Acid Protects Rat Chondrocytes Against IL-1β-Induced ECM Degradation by Activating Autophagy via PI3K/AKT/mTOR Pathway and Ameliorates Rat Osteoarthritis. Drug Des. Dev. Ther..

[32] García-Bonilla M., Ojeda-Pérez B., García-Martín M.L., Muñoz-Hernández M.C., Vitorica J., Jiménez S., Cifuentes M., Santos-Ruíz L., Shumilov K., Claros S. (2020). Neocortical tissue recovery in severe congenital obstructive hydrocephalus after intraventricular administration of bone marrow-derived mesenchymal stem cells. Stem Cell Res. Ther..

[33] Sena P., Mancini S., Pedroni M., Reggiani Bonetti L., Carnevale G., Roncucci L. (2022). Expression of Autophagic and Inflammatory Markers in Normal Mucosa of Individuals with Colorectal Adenomas: A Cross Sectional Study among Italian Outpatients Undergoing Colonoscopy. Int. J. Mol. Sci..

[34] Wang Z., Liu N., Liu K., Zhou G., Gan J., Wang Z., Shi T., He W., Wang L., Guo T. (2015). Autophagy mediated CoCrMo particle-induced peri-implant osteolysis by promoting osteoblast apoptosis. Autophagy.

[35] Qu Y., Cao J., Wang D., Wang S., Li Y., Zhu Y. (2022). 14,15-Epoxyeicosatrienoic Acid Protect Against Glucose Deprivation and Reperfusion-Induced Cerebral Microvascular Endothelial Cells Injury by Modulating Mitochondrial Autophagy via SIRT1/FOXO3a Signaling Pathway and TSPO Protein. Front. Cell. Neurosci..

[36] Haimovici A., Höfer C., Badr M.T., Bavafaye Haghighi E., Amer T., Boerries M., Bronsert P., Glavynskyi I., Fanfone D., Ichim G. (2022). Spontaneous activity of the mitochondrial apoptosis pathway drives chromosomal defects, the appearance of micronuclei and cancer metastasis through the Caspase-Activated DNAse. Cell Death Dis..

[37] Thirusangu P., Pathoulas C.L., Ray U., Xiao Y., Staub J., Jin L., Khurana A., Shridhar V. (2021). Quinacrine-Induced Autophagy in Ovarian Cancer Triggers Cathepsin-L Mediated Lysosomal/Mitochondrial Membrane Permeabilization and Cell Death. Cancers.

[38] Mandhair H.K., Arambasic M., Novak U., Radpour R. (2020). Molecular modulation of autophagy: New venture to target resistant cancer stem cells. World J. Stem Cells.

[39] Wu Y.H., Mo S.T., Chen I.T., Hsieh F.Y., Hsieh S.L., Zhang J., Lai M.Z. (2022). Caspase-8 inactivation drives autophagy-dependent inflammasome activation in myeloid cells. Sci. Adv..

[40] Liu B., Lu Y., Wang Y., Ge L., Zhai N., Han J. (2019). A protocol for isolation and identification and comparative characterization of primary osteoblasts from mouse and rat calvaria. Cell Tissue Bank..

[41] Chen J., Jin W., Zhong C., Cai W., Huang L., Zhou J., Peng H. (2024). Human umbilical cord mesenchymal stem cells promote steroid-induced osteonecrosis of the femoral head repair by improving microvascular endothelial cell function. Aging.

[42] Peng P., Wang X., Qiu C., Zheng W., Zhang H. (2023). Extracellular vesicles from human umbilical cord mesenchymal stem cells prevent steroid-induced avascular necrosis of the femoral head via the PI3K/AKT pathway. Food Chem. Toxicol..

[43] Sun H., Zhang W., Yang N., Xue Y., Wang T., Wang H., Zheng K., Wang Y., Zhu F., Yang H. (2021). Activation of cannabinoid receptor 2 alleviates glucocorticoid-induced osteonecrosis of femoral head with osteogenesis and maintenance of blood supply. Cell Death Dis..

[44] Nie N., Huo J., Sun S., Zuo Z., Chen Y., Liu Q., He S., Gao S., Zhang H., Zhao N. (2023). Genome-wide characterization of the PIFs family in sweet potato and functional identification of *IbPIF3.1* under drought and *Fusarium* wilt stresses. Int. J. Mol. Sci..

[45] Jia X.H., Geng L.Y., Jiang P.P., Xu H., Nan K.J., Yao Y., Jiang L.L., Sun H., Qin T.J., Guo H. (2020). The biomarkers related to immune related adverse events caused by immune checkpoint inhibitors. J. Exp. Clin. Cancer Res..

[46] Yao X., Yu S., Jing X., Guo J., Sun K., Guo F., Ye Y. (2020). PTEN inhibitor VO-OHpic attenuates GC-associated endothelial progenitor cell dysfunction and osteonecrosis of the femoral head via activating Nrf2 signaling and inhibiting mitochondrial apoptosis pathway. Stem Cell Res. Ther..

[47] Li W., Wei C., Xu L., Yu B., Chen Y., Lu D., Zhang L., Song X., Dong L., Zhou S. (2021). Schistosome infection promotes osteoclast-mediated bone loss. PLoS Pathog..

[48] Zhang W., Chen L., Wu J., Li J., Zhang X., Xiang Y., Li F., Wu C., Xiang L., Ran Q. (2019). Long noncoding RNA TUG1 inhibits osteogenesis of bone marrow mesenchymal stem cells via Smad5 after irradiation. Theranostics.

[49] Nishikawa G., Kawada K., Nakagawa J., Toda K., Ogawa R., Inamoto S., Mizuno R., Itatani Y., Sakai Y. (2019). Bone marrow-derived mesenchymal stem cells promote colorectal cancer progression via CCR5. Cell Death Dis..

[50] Yoon P.W., Kang J.Y., Kim C.H., Lee S.J., Yoo J.J., Kim H.J., Kang S.K., Min J.H., Yoon K.S. (2021). Culture-Expanded Autologous Adipose-Derived Mesenchymal Stem Cell Treatment for Osteonecrosis of the Femoral Head. Clin. Orthop. Surg..

[51] Yang G., Shao J., Lin J., Yang H., Jin J., Yu C., Shen B., Hu X., Si H., Li X. (2021). Transplantation of Human Umbilical Cord Blood-Derived Mesenchymal Stem Cells Improves Cartilage Repair in a Rabbit Model. Biomed. Res. Int..

[52] Menasché P. (2018). Cell therapy trials for heart regeneration—*Lessons* learned and future directions. Nat. Rev. Cardiol..

[53] Houdek M.T., Wyles C.C., Martin J.R., Sierra R.J. (2014). Stem cell treatment for avascular necrosis of the femoral head: Current perspectives. Stem Cells Cloning Adv. Appl..

[54] Benignus C., Lüring C., Beckmann J. (2020). Core decompression (“conventional method”) in atraumatic osteonecrosis of the hip. Oper. Orthop. Traumatol..

[55] Bougioukli S., Sugiyama O., Pannell W., Ortega B., Tan M.H., Tang A.H., Yoho R., Oakes D.A., Lieberman J.R. (2018). Gene Therapy for Bone Repair Using Human Cells: Superior Osteogenic Potential of Bone Morphogenetic Protein 2-Transduced Mesenchymal Stem Cells Derived from Adipose Tissue Compared to Bone Marrow. Hum. Gene Ther..

[56] Jiao F., Meng L., Du K., Li X. (2025). The autophagy-lysosome pathway: A potential target in the chemical and gene therapeutic strategies for Parkinson’s disease. Neural Regen. Res..

[57] Fan T., Yang S., Huang Z., Wang W., Guo X., Pan S., Zhang B., Xu Y., Fang Y., Mao Z. (2020). Autophagy decreases alveolar epithelial cell injury by regulating the release of inflammatory mediators. J. Cell. Physiol..

[58] Han D., Yang B., Olson L.K., Greenstein A., Baek S.H., Claycombe K.J., Goudreau J.L., Yu S.W., Kim E.K. (2010). Activation of autophagy through modulation of 5′-AMP-activated protein kinase protects pancreatic β-cells from high glucose. Biochem. J..

[59] Song J., Liu J., Cui C., Hu H., Zang N., Yang M., Yang J., Zou Y., Li J., Wang L. (2023). Mesenchymal stromal cells ameliorate diabetes-induced muscle atrophy through exosomes by enhancing AMPK/ULK1-mediated autophagy. J. Cachexia Sarcopenia Muscle.

[60] Park H.J., Shin J.Y., Kim H.N., Oh S.H., Lee P.H. (2014). Neuroprotective effects of mesenchymal stem cells through autophagy modulation in a parkinsonian model. Neurobiol. Aging.

